# Berry and phenology-related traits in grapevine (*Vitis vinifera *L.): From Quantitative Trait Loci to underlying genes

**DOI:** 10.1186/1471-2229-8-38

**Published:** 2008-04-17

**Authors:** Laura Costantini, Juri Battilana, Flutura Lamaj, Girolamo Fanizza, Maria Stella Grando

**Affiliations:** 1 Genetics and Molecular Biology Department, IASMA Research Center, Via E. Mach 1, 38010 San Michele all'Adige (TN), Italy; 2 DIBCA, University of Bari, Via Amendola 165/A, 70100 Bari, Italy

## Abstract

**Background:**

The timing of grape ripening initiation, length of maturation period, berry size and seed content are target traits in viticulture. The availability of early and late ripening varieties is desirable for staggering harvest along growing season, expanding production towards periods when the fruit gets a higher value in the market and ensuring an optimal plant adaptation to climatic and geographic conditions. Berry size determines grape productivity; seedlessness is especially demanded in the table grape market and is negatively correlated to fruit size. These traits result from complex developmental processes modified by genetic, physiological and environmental factors. In order to elucidate their genetic determinism we carried out a quantitative analysis in a 163 individuals-F_1 _segregating progeny obtained by crossing two table grape cultivars.

**Results:**

Molecular linkage maps covering most of the genome (2n = 38 for *Vitis vinifera*) were generated for each parent. Eighteen pairs of homologous groups were integrated into a consensus map spanning over 1426 cM with 341 markers (mainly microsatellite, AFLP and EST-derived markers) and an average map distance between loci of 4.2 cM. Segregating traits were evaluated in three growing seasons by recording flowering, veraison and ripening dates and by measuring berry size, seed number and weight. QTL (Quantitative Trait Loci) analysis was carried out based on single marker and interval mapping methods. QTLs were identified for all but one of the studied traits, a number of them steadily over more than one year. Clusters of QTLs for different characters were detected, suggesting linkage or pleiotropic effects of loci, as well as regions affecting specific traits. The most interesting QTLs were investigated at the gene level through a bioinformatic analysis of the underlying Pinot noir genomic sequence.

**Conclusion:**

Our results revealed novel insights into the genetic control of relevant grapevine features. They provide a basis for performing marker-assisted selection and testing the role of specific genes in trait variation.

## Background

Control of the main phenological events, berry size and aromatic composition are target traits for viticulturists and wine makers. Additionally, in the table grape market there is an increasing demand for seedless varieties.

Phenology is the most important attribute involved in the adaptation of grapevine, as other crops, to its growing environment and to climatic changes [1,2]. It is a complex trait, which results from the interaction of various developmental quantitative characters such as flowering, veraison and fruit ripening.

The genetic control of flowering has been extensively studied in the model plant *Arabidopsis thaliana *[3,4]. On the other hand, research in woody species like grapevine is made difficult by the long juvenile or non-flowering period of seed-grown plants, by the large size of adult trees, and by the annual occurrence of flowers. Despite the conservation of several flowering pathways among plants, there may be major differences in the mechanisms of flower induction in the long-day plant *Arabidopsis *compared with most short-day plants and woody perennials. Similar genes may be involved, but it is highly probable that they are regulated in a different manner or have different downstream effects than in *Arabidopsis*. Flowering in *Vitis vinifera *differs significantly from that in *Arabidopsis *in having distinct juvenile and adult periods during development; this process takes 2 years in adult grapevine plants and is mediated by a peculiar meristematic structure (uncommitted primordium) at the origin of both tendrils and inflorescences [5]. The environmental and endogenous influences on grapevine flowering are different from those acting on *Arabidopsis*. In *Arabidopsis*, flowering is stimulated by gibberellins (GAs), long days and vernalization. In grapevine the variables that promote flowering are light intensity, high temperature and GA inhibitors, while vernalization and long days do not have a marked effect. Although much work has been devoted to the physiology of grape flowering in order to forecast crop and to increase or decrease yield, very little is known about the underlying molecular mechanisms. In the last years the grapevine orthologs of some *Arabidopsis *flowering genes have been cloned and characterized: *VvMADS1*, an *AGAMOUS*/*SHATTERPROOF *homologue [6]; *VvMADS2 *and *VvMADS4*, related to the *SEPELLATA *genes, *VvMADS3*, homologous to *AGAMOUS-LIKE6 *and *13*, and *VvMADS5*, homologous to *AGAMOUS-LIKE11 *[7,8]; *VFL*, the homologue of *LEAFY *[8,9]; *VAP1 *and *VFUL-L*, respectively homologous to *APETALA1 *and *FRUITFULL*-like [8,10]; *VvTFL1*, the homologue of *TERMINAL FLOWER1 *[8,11,12]; *VvFT *and *VvMADS8*, respectively homologous to *FLOWERING TIME *and *SUPPRESSOR OF OVEREXPRESSION OF CONSTANS1 *[12,13]; *VvMFT*, the homologue of *MOTHER OF FT AND TFL1 *[12].

Unique features characterize also the process of fruit development in grapevine. Fruit ripening is a highly programmed event relying on the coordinated activation of numerous genes mainly controlling cell-wall composition, sugar and water import, organic acid metabolism and storage, anthocyanin synthesis and response towards biotic or abiotic stress [14,15].

Two kinds of seedlessness exist in grapevine [16]: parthenocarpy (i. e. in Corinth cultivars) and stenospermocarpy (i. e. in Thompson cultivars). Parthenocarpic fruits are seedless because the ovary is able to develop without ovule fertilization, thanks to the stimulus of pollination. The small size of berries from parthenocarpic grapes makes them suitable only for the production of raisins. In stenospermocarpic varieties pollination and fertilization occur as normal, but the embryo and/or endosperm abort two to four weeks after fertilization; as a result, seed development ceases (leaving only partially formed seeds or seed traces), while the ovary wall pericarp continues to grow and originates berries which still have a size compatible with commercial requirements for fresh fruit consumption. Different hypothesis have been proposed for the genetic control of seedlessness [17], the predominant one suggesting the involvement of three independent and complementary recessive genes regulated by a dominant gene, later named *SdI *(*Seed development Inhibitor*) [18], which inhibits seed development. Recently differential expression analysis between a seeded and a seedless Thompson line identified a gene coding for the chloroplast chaperonin 21 (ch-Cpn21), whose silencing in tobacco and tomato fruits resulted in seed abortion [19]. The authors concluded that the ch-Cpn21 protein is essential for grape seed development.

In grapevine an undesired negative correlation exists between seedlessness and berry size [20], since seed tissues supply important hormones for fruit development [21,22]. However additional mechanisms could be involved in the regulation of berry size. The monogenic *fleshless berry *(*flb*) mutation in *Vitis vinifera *L. cv Ugni Blanc early after fertilization impairs the differentiation and division of the most vacuolated cells in the inner mesocarp that forms the flesh, resulting in a 10-fold reduction in fruit weight [23]. The defect is not simply a deficiency in plant growth regulator levels and does not show any obvious relationship with fertility, seed size or number.

All the above traits are under strict hormonal control. It has been suggested that grapevine flowering is regulated by the gibberellin:cytokinin balance. Gibberellins inhibit inflorescence and promote tendril development [24], while cytokinins can result in the production of inflorescences from tendril meristems [25]. Also fruit ripening is likely triggered by a number of hormonal factors. Despite grapes have been classified as non-climacteric fruits, evidence of a transient increment in endogenous ethylene level prior to veraison suggested that ethylene perception is required for at least the increase of berry diameter, the decrease of berry acidity and the accumulation of anthocyanins in the ripening berries [26]. Other plant hormones, such as auxin and abscissic acid, have been proposed to control grape ripening. Grape berry ripening may be initiated by the combination of a decline in auxin level coupled with an increment in abscissic acid level [27,28]. Moreover, Symons et al. [29] demonstrated that it is associated also with a rise in endogenous brassinosteroids. Finally, gibberellins are likely to take a prominent part in seedlessness [17,30,31], possibly in association with other growth substances, like auxins [32,33], or ethylene [34]. Treatments with gibberellins, besides delaying ripening, are effective in the promotion of seedlessness in seeded grapes, the suppression of vestigial seed development in normally seedless grapes, the increase of berry and cluster size and the decrease of cluster compactness [35,36].

The aim of this work was to investigate the genetic determinism of flowering and fruit maturation timing, berry size and seed content in grapevine. Linkage maps containing microsatellite, AFLP and EST-based markers were developed for a table grape segregating F_1 _progeny and used to perform quantitative analysis in combination with phenotypic data collected over three years. The most significant QTLs were further analyzed by exploiting the recently published Pinot noir genomic sequence [37,38].

## Results

### Markers

The number and segregation type of the markers used to generate the maps of Italia and Big Perlon are shown in Table 1. The 112 microsatellites yielded 114 markers, as in 2 cases (VVIQ22b and VMC2B5) segregation pattern was consistent with the presence of a null allele in Italia (a0xab) and re-coding was adopted. The 20 MseI/EcoRI combinations provided a total number of 1380 AFLP markers (minimum 42 and maximum 106 per primer combination). Two hundred seventy-five of them were polymorphic, resulting in a polymorphism percentage comprised between 13 and 32 (mean value: 20). Fourteen AFLP markers were removed because of inconsistencies in the phase chosen by JoinMap, leaving a total of 261 loci in the final mapping data set. The SCAR marker SCC8, berry colour and seedlessness segregated 1:1 in the progeny. Thirty-five markers derived from ESTs were mapped after SSCP and minisequencing analysis [39].

**Table 1:** Number and segregation type of the markers analyzed in the progeny Italia × Big Perlon

**Segregation**	**Type**	**SSRs**	**AFLPs**	**EST-based markers**	**SCARs**	**Morphological markers**
<abxcd>	1:1:1:1	21				
<efxeg>	1:1:1:1	37		1		
<hkxhk>	1:2:1 or 3:1	7	64	8		
<lmxll>	1:1	34	120	14	1	
<nnxnp>	1:1	15	77	12		2
Total	413	114	261	35	1	2

### Genetic maps

**Figure 1:**
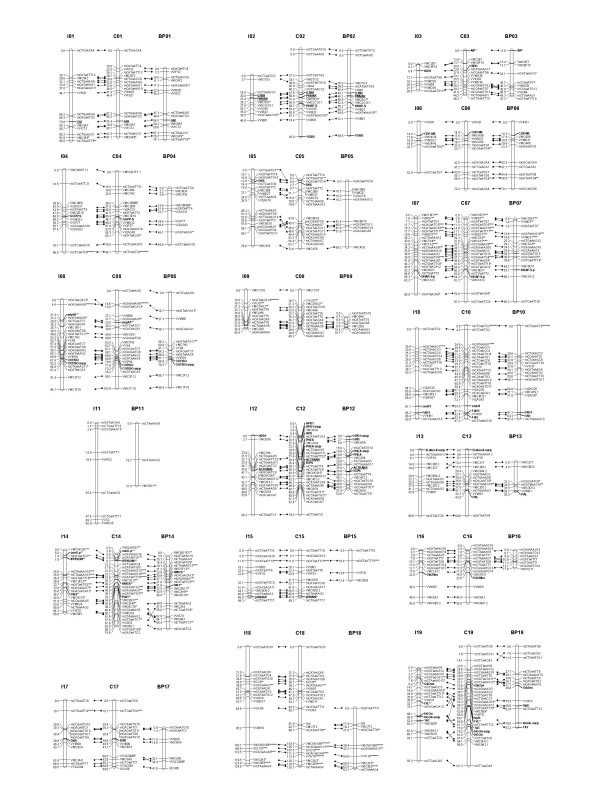
**Linkage map of *Vitis vinifera *Italia × Big Perlon**. Linkage groups are numbered according to [40]. For each linkage group, the parental maps are shown on the left (Italia) and right (Big Perlon) and the consensus map is in the centre. Markers common between parental and consensus maps are indicated by lines. Distorted markers have an asterisk showing the level of distortion (* = P ≤ 0.1, ** = P ≤ 0.05, *** = P ≤ 0.01; **** = P ≤ 0.005; ***** = P ≤ 0.001; ****** = P ≤ 0.0005; ******* = P ≤ 0.0001). Underlined markers are EST-based markers analyzed in the progeny Moscato bianco × *Vitis riparia *and mapped for synteny in the maps of Italia and Big Perlon. Distances of markers from the top are indicated on the left in cM Kosambi.

For the maternal map 98 SSRs, 154 AFLPs, 23 EST-based markers and 1 SCAR marker (SCC8) were assembled into 19 linkage groups spanning 1353 cM of map distance with an average interval length of 4.9 cM; the paternal map was established on 80 SSRs, 107 AFLPs, 21 EST-based markers and 2 morphological markers (colour and seedlessness, *SdI*) which were positioned on 19 linkage groups and covered altogether 1130 cM with an average interval length of 5.4 cM (Figure 1 and Table 2).

**Table 2:** Summarizing outline of Italia, Big Perlon and consensus maps

	**Italia**	**Big Perlon**	**Consensus**
N. of analyzed markers	308	245	370
N. of mapped markers	276	210	341
SSRs	98	80	107
AFLPs	154	107	196
EST-based markers	23	21	35
SCARs	1	-	1
morphological markers	-	2	2
N. of ungrouped markers	20	25	-
N. of unpositioned markers	12	10	29
N. of linkage groups (LG)	19	19	18
Mean number of markers/LG	15	11	19
N. of markers/LG range	7–22	3–21	13–29
Total length (cM)	1353	1130	1426
Mean LG length (cM)	71	60	79
LG length range (cM)	26–125	11–99	40–126
Average map distance between loci (cM)	4.9	5.4	4.2
N. of gaps between 20 and 30 cM	10	4	7
N. of gaps > 30 cM	1	1	1

"Ungrouped" markers could not be assigned to any linkage group, "unpositioned" markers could be assigned but not placed on the maps because of insufficient linkage to the other loci or location conflicts.

Additional 12 and 10 markers have been attributed respectively to Italia and Big Perlon linkage groups in the absence of a definite linear order. Some loci could not be assigned to any linkage group; a possible explanation is that they are located in regions of the genome not yet covered by the present maps. For the Italia map the average size of linkage groups was 71 cM, ranging from 26 to 125 cM; for the Big Perlon map the average size was 60 cM, ranging from to 11 to 99 cM. The total number of positioned markers per linkage group was between 7 (LG 6) and 22 (LGs 7 and 8) for Italia and between 3 (LG 11) and 21 (LG 14) for Big Perlon. Marker-free regions longer than 20 cM were found in 11 Italia linkage groups and 5 Big Perlon linkage groups (Table 2). The consensus map consisted of 341 markers mapped on 18 linkage groups (LG 11 was excluded), covering 1426 cM with an average interval length of 4.2 cM. The average size of linkage groups was 79 cM, ranging from 40 to 126 cM; the total number of positioned markers per linkage group was between 13 (LGs 6, 9 and 15) and 29 (LG 19); marker-free regions longer than 20 cM were found in 8 linkage groups (Figure 1 and Table 2). Five further EST-based markers, monomorphic in the Italia × Big Perlon progeny, were analyzed in a population derived from the cross between Moscato bianco and *Vitis riparia *and then mapped for synteny (Figure 1), as already reported in literature [41].

The major genes for berry colour and seedlessness were located as Mendelian markers respectively on LGs 2 and 18 (Figure 1), in agreement with [42-44].

Pronounced clustering of any marker type was not evident in the parental maps. AFLP marker distribution was analyzed by calculating the Pearson correlation coefficient between the number of AFLP markers in the linkage groups and the size of the linkage groups [45]. The correlation was significant (at the 0.01 level for Italia and 0.05 level for Big Perlon), indicating that AFLP markers are randomly distributed. Chi-square analysis revealed a distorted segregation ratio (P ≤ 0.05) for 17.4% of the markers polymorphic in Italia and 16.9% of the markers polymorphic in Big Perlon. This amount of distortion is comparable (on the whole, slightly higher) to the percentages already reported for grapevine [40,42,43,46-51].

The frequency of distorted alleles was faintly higher for the female parent: respectively 18.7% and 18.5% of the markers segregating 1:1 showed segregation distortion in Italia and in Big Perlon; among loci for which segregation distortion could be tested separately in both parents, 4 loci segregating 1:1.1:1 (VMC7A4, VMCNG1E1, VVMD7 and VVMD31) showed distorted segregation only in Italia and 2 loci segregating 1:1:1.1 (VMC1E12 and VMCNG1B9) showed distorted segregation in both parents. As already reported by other authors [42,43,47,49-51], most of the distorted markers clustered together on some linkage groups (in our case LGs 7, 14 and 18). Interestingly, markers with skewed segregation were reported on LG14 also for the crosses Chardonnay × Bianca [49,52] and Ramsey × Riparia Gloire [51] and on LG18 in the map of Autumn Seedless [43]. Only LG7 was unidirectional in bias (all markers showed an excess of the female allele), while LGs 14 and 18 were bi-directional.

Marker order was generally consistent between homologs from the parental and the consensus maps, thus suggesting not too different recombination frequencies between Italia and Big Perlon; most of the inversions present on several linkage groups occurred between closely linked markers. A simple correlation between distorted markers and rearrangements does not seem to exist as only a few small inversions may be accounted for by segregation distortion, whereas some linkage groups (LGs 7 and 18, for example) have many distorted markers and no rearrangements.

When comparing our maps to five other published maps with high numbers of SSRs [40,43,48,50,51] and to the first integrated map of grapevine [49], complete agreement exists with respect to linkage groups, while marker order is similar but less consistent. There are discrepancies in marker order between our consensus map and [40] (84 shared SSRs) for the linkage groups 2, 4, 8, 18 and 19, [43] (64 shared SSRs) for the linkage groups 8, 10 and 19, [48] (81 shared SSRs) for the linkage groups 3, 4, 5, 6, 7, 12 and 18, [50] (85 shared SSRs) for the linkage groups 3, 8 and 18, and finally [51] (55 shared SSRs) for the linkage groups 7, 10, 18 and 19. These inconsistencies reflect the limitations inherent in the small population sizes on which the maps are based (from 96 to 188 plants, respectively in [40] and [51]) and the statistical method used to perform linkage analysis. Our map shares 109 microsatellites with the composite map reported in [49] and shows discrepancies in marker order for the groups 3, 4, 6, 9, 10, 13, 18 and 19. In most cases they are small inversions in regions where groups of loci with local order unsure at LOD 2.0 were mapped in [49].

### Comparison of parental meiotic recombination rates

Parental recombination rates were compared at 71 intervals between common markers, covering twelve out of nineteen linkage groups. Recombination was slightly higher in Italia (0.1978 vs 0.1944), although not statistically significant at the 0.05 level based on a Z test (1.9600). This observation is in agreement with what reported to date on the effect of sex on recombination rate in grape [42,46,48,51,53]. Among the 71 pairs of linked markers for which parental recombination rates were compared, twelve showed statistically significant (P ≤ 0.05) differences.

Recombination was higher in the maternal parent for five pairs (VVIP04-VMC2F12, VMC2F12-VMC7H2, VMC2F12-VVS4 in group 8, VMC8G6-VMC2H4 in group 12 and VMC6C10-VVIS70 in group 14) and higher in the paternal parent for seven pairs (VMC8F10-VVIN54, VMC8F10-VVMD36, VMC2E7-VVIN54, VMC2E7-VVMD36 in group 3, VMC2H4-VMC4F3.1 in group 12 and VMC6C10-VMCNG1E1, VMCNG1E1-VMC1E12 in group 14). The observation that among the three linkage groups with the highest number of distorted markers (LGs 7, 14 and 18) only LG14 showed statistically significant differences in parental recombination rates seems to suggest that only in some cases differences in recombination rates may account for segregation distortion.

In conclusion, the greater length of the Italia map with respect to that of Big Perlon is presumably due to a greater number of markers rather than to differences in the recombination rate between parents.

### Genome length

Genome length estimates differed between paternal and maternal data sets (Table 3). Their average value was smaller when considering all mapped markers (1693 cM) with respect to that obtained when excluding all AFLPs (1908 cM), opposite to what was observed by [42]. However, like in [42], confidence intervals were larger when excluding AFLPs. Mean observed genome coverage with all markers was 73.2% versus an expected coverage of 92.6% according to [54] and 89.6% according to [55], whereas mean observed genome coverage in absence of AFLPs was 42.7% versus an expected coverage of 79.9% according to [54] and 75.6% according to [55].

**Table 3:** Estimated genome length, expected and observed map coverage with Kosambi mapping function

	**Italia**	**Big Perlon**
**With AFLPs**		
Number of markers (N)	276	210
Number of linkages with LOD ≥ 5 (K)	873	534
Maximum observed map distance (X)	20.6	19.4
Estimated genome length (cM)	1791	1595
Confidence interval (95%)	1680–1918	1470–1742
Expected genome map coverage [54]	94.6%	90.5%
Expected genome map coverage [55]	92.1%	87.0%
Observed genome map coverage	75.5%	70.9%
**Without AFLPs**		
Number of markers (N)	120	101
Number of linkages with LOD ≥ 5 (K)	212	174
Maximum observed map distance (X)	29.0	29.0
Estimated genome length (cM)	1953	1683
Confidence interval (95%)	1722–2257	1466–1977
Expected genome map coverage [54]	80.5%	79.3%
Expected genome map coverage [55]	76.0%	75.1%
Observed genome map coverage	44.5%	40.9%

The estimated genome sizes of Italia (1791 cM) and Big Perlon (1595 cM) are slightly greater than those reported by [43,51], comparable to those reported by [40,42,44] and much smaller than those reported by [48]. This last discrepancy may be due to the size of the largest marker gap, as genome size estimations based on Hulbert's equation inflate with higher maximum observed map distances (X). [48] reported maximum distances between markers of 49.0 and 44.7 cM, while X values were 20.6 and 19.4 for Italia and Big Perlon maps, respectively. Observed genome coverage of Italia and Big Perlon maps was among the highest accounted for grape.

### Phenotypic data

**Figure 2:**
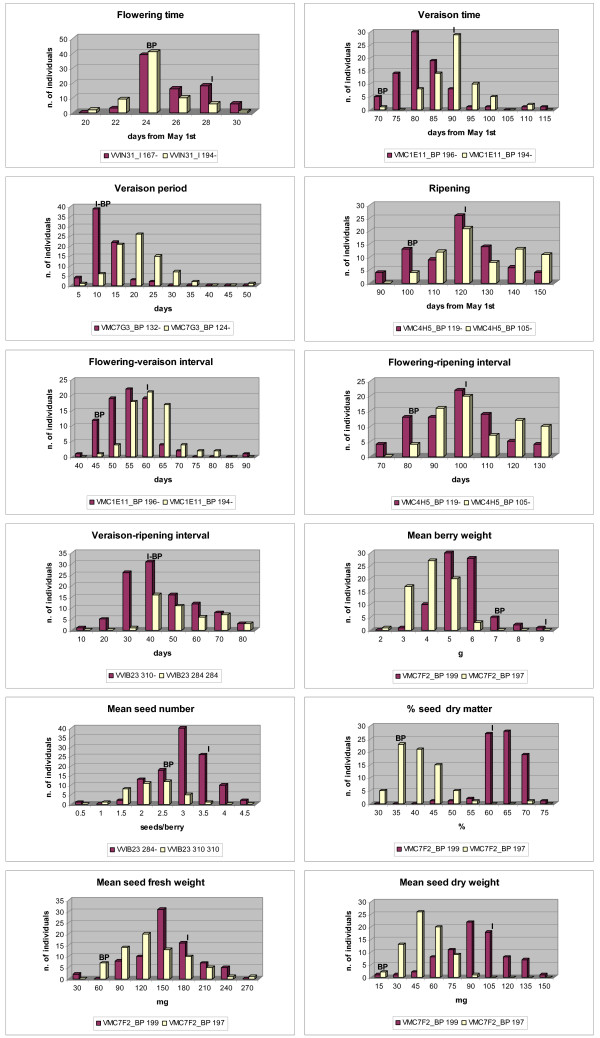
**Distribution of phenotypic traits in 2003**. The microsatellite marker explaining the highest proportion of variability for each trait (Table 5) was used as dividing criterium to identify two subpopulations with different alleles. Allele sizes are reported in the legend (I = Italia, BP = Big Perlon).

Phenotypic data distributions, which are shown in Figure 2 for year 2003, were very similar in the 3 years. A continuous variation, which is typical of quantitative traits, and a transgressive segregation were observed for all traits. The Kolmogorov-Smirnov test indicated departures from normality for flowering beginning, flowering end, flowering period, veraison beginning, veraison end, veraison-ripening interval and percentage of seed dry matter (P < 0.05 for at least two years).

Analysis of variance and Kruskal-Wallis test revealed a highly significant year effect (P < 0.01) for all the traits but the interval between flowering and veraison beginning. However, Spearman rank-order correlations between years turned out to be significant (at the 0.01 level) for all the traits, except for flowering period (data not shown). The lowest correlation was observed for flowering end date (r ranging from 0.315 to 0.489), the highest one for veraison beginning date (r ranging from 0.838 to 0.908). Several associations between traits within each year were revealed by Spearman rank-order correlation test. Many of them concerned the component variables of the same character; nevertheless correlations between different traits were also detected (Table 4): a positive correlation between veraison time (VB, VE, VT, F-V) and seed weight (% SDM, MSFW, MSDW); a positive correlation between veraison length (VP, V-R) and mean seed number (MSN); a positive correlation between mean berry weight (MBW) and seed weight (% SDM, MSFW, MSDW); a negative correlation between mean seed number (MSN) and seed dry matter (% SDM) and conversely a positive correlation between mean seed number (MSN) and mean seed fresh weight (MSFW).

**Table 4:** Phenotypic correlations between traits (Spearman correlation coefficient) averaged over three years

	**FE**	**FT**	**FP**	**VB**	**VE**	**VT**	**VP**	**R**	**F-V**	**F-R**	**V-R**	**MBW**	**MSN**	**SDM %**	**MSFW**	**MSDW**
**FB**	**0.72**	**0.92**	**-0.48**	**0.40**	**0.31**	**0.38**	NS**a-**	0.22b	NSa+	NS	NS**a**-	NS	NS	NS	NSa+	NS
**FE**		**0.92**	0.27b	**0.33**	**0.27**	**0.31**	NSa-	NS	NSa+	NS	NSa-	NS	NSa-	NS	NS	NS
**FT**			NS**a-**	**0.39**	**0.31**	**0.37**	NS**a-**	0.20b	NSa+	NS	NSa-	NS	NS	NS	NSa+	NS
**FP**				NSa-	NS	NS	NSa+	NS	NS	NS	NS	NS	NS	NS	NSa-	NS
**VB**					**0.70**	**0.90**	**-0.35**	**0.47**	**0.95**	**0.40**	-0.30b	NSa+	NS	0.25b	**0.33**b	**0.29**
**VE**						**0.94**	**0.50**b	**0.64**	**0.66**	**0.59**	0.31b	NSa+	NS	NSa+	0.24b	NS**a+**
**VT**							0.20c	**0.62**	**0.84**	**0.55**	c	NS**a+**	NS	NS**a+**	**0.30**b	0.28b
**VP**								**0.36**b	**-0.35**	**0.37**b	**0.55**	NS	0.19b	NS**a-**	NS	NSa-
**R**									**0.45**	**0.97**	**0.66**	NS**a+**	NS**a+**	NS	NS**a+**	NSa+
**F-V**										**0.44**	-0.28b	NSa+	NS	0.27b	**0.31**b	**0.28**
**F-R**											**0.70**	NSa+	NS**a+**	NS	NS**a+**	NSa+
**V-R**												NSa+	0.29b	NSa-	NS	NSa-
**MBW**													NS	**0.50**	**0.41**	**0.59**
**MSN**														**-0.26**	**0.36**	NS**a-**
**% SDM**															**0.34**b	**0.72**
**MSFW**																**0.77**

Boldface and normal font indicate respectively correlations which are significant at the 0.01 and 0.05 level; NS = not significant; a = correlation significant (+ = positive, - = negative) only in one year; b = correlation not significant in one year; c = contradictory result.

Correlations observed in only one year (in most cases 2004) as well as discordant correlations over different years (as found for veraison time) were not considered reliable.

### QTL analysis

QTL analysis was performed separately on the parental and consensus maps for three years (Table 5).

**Table 5:** Location, significance and effect of QTLs detected for phenology, berry size and seed content

**Trait**	**QTL position**						**LOD**	**LOD threshold**		**% var**	**KW sig**
					
	**LG**	**Map**	**Peak (cM)**	**Nearest marker**	**cM**	**Interval**		**α = 0.20**	**α = 0.05**		
**FT**	1	Ia	54.7	VVIS21	44.7	43.1–66.6	2.3, 3.2, 4.3	2.0	2.8	6.3, 11.7, 8.6	1, 1, 2
		Ib	88.2	mCTGeACC1		83.0–88.9	2.1, 4.7, 3.1	2.0	2.8	6.3, 11.5, 6.5	0, 3, 3
	1	Ca	54.4	VVIS21	44.4	44.3–65.0	2.3, 3.8, 4.6	2.2	3.2	7.8, 13.9, 9.1	1, 1, 2
		Cb	87.5	mCTGeACC1		82.8-b	-, 4.8, 3.1	2.2	3.2	-, 11.7, 6.6	-, 3, 3
	1	BPa	35.4	VVIS21	25.4	24.3–46.4	2.3, 3.1, 4.2	2.1	3.0	6.2, 11.4, 8.4	1, 1, 2
		BPb	67.3	mCTGeACC1		62.2–68.0	2.1, 4.7, 3.1	2.1	3.0	6.3, 11.5, 6.6	0, 3, 3
	2	I	35.0	VVIB23		31.9–36.5	3.4, 3.5, 8.2	2.0	2.9	9.1, 7.3, 16.1	3, 4, 7
	2	C	62.6	VVIB23		59.5–64.0	3.3, 3.6, 8.4	2.2	2.9	9.0, 7.7, 16.4	3, 4, 7
	2	BP	60.2	VVIB23		55.1–61.5	3.4, 3.5, 8.1	2.0	2.9	9.2, 7.4, 16.1	3, 4, 7
	6	I	5.0	VVIN31	t	t-9.5	4.1, 7.4, 6.8	1.8	2.6	13.8, 20.5, 15.5	3, 7, 5
	6	C	5.0	VVIN31	t	t-9.5	3.9, 7.2, 6.8	1.9	2.7	13.4, 19.9, 15.4	3, 7, 5
	6	BP	5.0	VVIN31	t	t-9.2	4.1, 7.4, 6.9	1.9	2.8	13.9, 20.8, 15.6	3, 7, 5
											
**VT**	2	I	30.1	VVIO55		27.0–31.9	3.5, 3.0, 5.6	2.3	3.5	6.6, 5.9, 12.6	0, 3, 1
	2	C	55.0	VMC2C10.1		52.0–55.2	3.5, 2.9, 5.6	2.6	4.0	6.6, 5.8, 12.6	0, 1, 1
	2	BP	52.5	VMC2C10.1		48.1–53.4	3.5, 2.9, 5.6	2.0	2.7	6.6, 5.8, 12.6	0, 1, 1
	6	I	17.6	VMC4G6		13.4–18.0	4.6, 4.9, -	1.6	2.4	9.0, 9.8, -	3, 4, -
	6	C	17.6	VMC4G6		13.5–17.9	4.8, 4.9, -	1.8	2.6	9.3, 9.9, -	3, 4, -
	6	BP	17.4	VMC4G6		13.4–17.7	4.8, 4.9, -	1.9	2.7	9.3, 9.9, -	3, 4, -
	16	I	17.8	VMC1E11		15.6–20.6	13.7, 9.7, 11.3	1.8	2.5	31.6, 21.1, 29.1	7, 7, 7
	16	C	16.9	VMC1E11	18.0	15.2–20.5	15.1, 9.6, 11.9	1.9	2.7	38.0, 24.1, 45.4	7, 7, 7
	16	BP	17.8	VMC1E11		14.3–17.8	14.0, 9.7, 11.5	1.5	2.2	32.1, 21.2, 29.1	7, 7, 7
											
**VP**	2	I	19.0	mCTGeACC2		3.6–20.2	13.6, 15.4, 7.0	2.1	3.9	41.8, 38.0, 44.2	7, 7, 7
	2	C	40.9	colour		40.2–45.9	14.0, 16.4, -	3.0	4.4	40.0, 39.8, -	7, 7, -
	2	BP	40.5	colour		39.8–44.4	13.9, 16.4, 4.3	2.0	2.7	38.9, 39.6, 15.8	7, 7, 7
											
**R**	6	I	19.6	VMC4H5		18.4–20.8	4.1, 3.5, -	1.7	2.5	17.2, 10.2, -	5, 2, -
	6	C	19.7	VMC4H5		18.5–21.0	4.1, 3.5, -	1.9	2.7	17.2, 10.2, -	5, 2, -
	6	BP	19.6	VMC4H5		18.3–20.8	4.1, 3.5, -	1.9	2.7	17.2, 10.2, -	5, 2, -
											
**F-V**	2	I	24.0	VMC5G7	24.2	20.4–24.8	7.7, 6.4, 5.7	2.5	3.9	18.7, 14.0, 12.6	6, 6, 4
	2	C	51.2	VMC5G7	51.4	47.1–51.8	7.7, 6.4, 5.8	2.8	4.1	18.4, 13.8, 12.7	6, 6, 4
	2	BP	45.5	VMC5G7	48.9	40.4–49.4	8.0, 6.5, 5.8	1.9	2.8	21.4, 15.6, 12.7	6, 6, 4
	6	I	17.6	VMC4G6		13.3–18.0	3.9, 4.0, -	1.7	2.5	8.5, 8.2, -	1, 1, -
	6	C	17.6	VMC4G6		13.3–18.0	3.9, 4.0, -	1.8	2.6	8.5, 8.2, -	1, 1, -
	6	BP	17.4	VMC4G6		13.3–17.8	3.9, 4.0, -	1.9	2.6	8.5, 8.2, -	1, 1, -
	16	I	17.8	VMC1E11		15.8–19.1	7.9, 7.3, 11.4	1.8	2.6	18.7, 15.5, 27.8	7, 7, 7
	16	C	18.0	VMC1E11		15.8–19.3	8.7, 7.2, 11.8	1.9	2.6	23.0, 15.4, 37.2	7, 7, 7
	16	BP	17.8	VMC1E11		15.0–17.8	7.9, 7.2, 11.4	1.4	2.2	18.7, 15.4, 27.8	7, 7, 7
											
**F-R**	6	I	19.6	VMC4H5		18.3–21.0	3.6, 3.1, -	1.7	2.6	15.3, 9.1, -	4, 2, -
	6	C	19.7	VMC4H5		18.3–21.2	3.6, 3.1, -	1.8	2.5	15.3, 9.1, -	4, 2, -
	6	BP	19.6	VMC4H5		18.1–21.0	3.6, 3.1, -	1.9	2.6	15.3, 9.1, -	4, 2, -
											
**V-R**	2	I	35.0	VVIB23#		29.9–35.7	3.3, 6.6, 6.0	2.0	3.0	15.4, 18.0, 20.7	2, 7, 7
	2	C	62.6	VVIB23#		57.3–63.3	3.4, 6.5, 5.9	2.2	3.1	14.6, 17.8, 19.9	2, 7, 7
	2	BP	60.2	VVIB23#		55.1–60.8	3.7, 6.6, 6.2	2.0	2.8	15.9, 18.1, 21.7	2, 7, 7
	12*	I	18.7	VMC2H4	23.8	7.8–29.5	3.2, -, 2.6	2.0	2.8	16.7, -, 10.4	3, -, 1
	12*	C	21.9	VMC2H4		21.4–28.5	3.3, -, 2.8	1.9	2.7	13.5, -, 9.0	3, -, 3
	12*	BP	18.8	PHEA-sscp		16.3–19.2	3.5, -, 2.5	1.9	2.6	16.8, -, 9.1	0, -,0
											
**MBW**	1	C	18.9	mCACeATC4		t-19.5	3.2, 2.6, 5.4	2.2	3.1	10.7, 4.7, 17.5	1, 2, 4
	1	BP	t	mCACeATC4		t-0.9	-, 2.4, 3.7	2.0	2.8	-, 4.6, 9.1	-, 2, 4
	12	C	29.8	mCTGeAAG5		22.8–29.8	3.4, 3.2, 3.9	1.9	2.8	8.4, 5.6, 8.8	2, 2, 6
	12	BP	28.5	mCTGeAAG5		20.6–28.6	2.4, 3.2, 3.7	1.9	2.8	5.1, 5.7, 8.0	1, 2, 6
	18	C	81.9	SdI		74.5–81.9	14.2, 19.4, 11.5	2.5	3.3	41.7, 43.1, 32.6	7, 7, 7
	18	BP	17.4	SdI		13.8–17.4	11.8, 18.3, 9.9	1.9	2.6	29.6, 40.8, 27.2	7, 7, 7
											
**MSN**	2	I	35.0	VVIB23		31.6–35.6	6.2, 8.5, -	1.9	2.6	19.6, 22.9, -	7, 7, -
	2	C	62.6	VVIB23		60.8–63.0	6.3, 8.5, -	2.1	2.9	19.9, 22.9, -	7, 7, -
	2	BP	60.2	VVIB23		58.7–60.7	6.3, 8.5, -	2.0	2.7	19.8, 22.9, -	7, 7, -
											
**% SDM **	18	BP	17.4	SdI		13.1–17.5	65.9, 61.7, 59.2	2.1	3.6	90.0, 86.5, 91.4	7, 7, 7
											
**MSFW**	6*	BP	42.3	mCACeACA4		39.4–48.3	3.4, -, 4.2	1.9	2.7	5.3, -, 13.2	0, -, 4
	6	C	47.2	mCTCeACA1		37.4–48.3	4.3, 2.1, 5.8	1.8	2.5	11.3, 3.5, 21.4	1, 1, 4
	10	I	43.3	mCTAeAAG10		39.0–53.0	2.6, 3.3, -	2.0	2.7	9.4, 10.0, -	0, 0, -
	10	C	47.2	mCTAeAAG10#		46.9–55.6	4.4, 6.4, '-	2.3	3.0	36.3, 13.4, -	2, 0, -
	10	BP	50.0	mCTGeATT18	50.6	44.8–55.7	2.9, 4.2, 2.2	2.0	2.7	7.7, 12.1, 9.8	2, 3, 0
	13	I	62.6	mCATeATG9#	b	42.5–b	3.5, 3.9, -	1.9	2.7	14.6, 14.2, -	1, 3, -
	13	C	b	mCATeATG9#		52.0–b	3.8, 4.4, -	2.3	3.0	8.8, 15.7, -	0, 3, -
	13	BP	25.5	VMC3D12#		25.1–29.8	3.3, 3.4, -	1.7	2.4	7.2, 7.8, -	1, 2, -
	15	I	5.8	mCACeACA10#		5.5–13.5	2.1, 2.6, 3.5	1.9	2.6	7.5, 6.7, 13.0	0, 0, 5
	18	C	80.1	SdI	81.9	65.0–82.0	10.7, 5.5, 6.4	2.4	3.2	27.5, 15.8, 25.7	7, 4, 6
	18	BP	17.4	SdI		8.7–17.7	10.3, 3.9, 5.9	1.9	2.7	26.3, 13.8, 24.8	7, 4, 6
											
**MSDW**	2*	I	35.0	VVIB23		29.8–36.7	3.3, -, 2.9	1.8	2.6	10.5, -, 10.8	6, -, 2
	2*	C	62.6	VVIB23		60.4–63.2	8.9, -, 2.3	2.2	8.0	4.6, -, 3.7	6, -, 2
	15	I	5.8	mCACaACA10#		5.2–12.3	-, 2.9, 2.2	1.8	2.6	-, 10.8, 8.2	-, 0, 2
	15	C	5.7	mCACeACA10#		5.5–9.4	-, 2.2, 3.2	1.8	2.8	-, 3.1, 5.4	-, 0, 2
	18	C	80.1	SdI	81.9	72.0–81.4	55.5, 27.5, 19.8	2.2	3.4	73.8, 57.1, 49.3	7, 7, 7
	18	BP	15.0	SdI	17.4	12.9–17.4	54.5, 25.6, 19.0	2.0	2.8	75.0, 62.1, 49.4	7, 7, 7

LG = linkage group; Map = map in which the QTL was identified (I for Italia, C for consensus, BP for Big Perlon); Peak = QTL position as estimated by the cM distance of the local LOD maximum from the top of the linkage group, with 't' for top and 'b' for bottom of linkage group; Nearest marker = marker nearest to the QTL position; Interval = 1-LOD confidence interval of QTL position in cM; # = LOD peak position and confidence interval were not exactly the same in different years; LOD = LOD value at QTL position; LOD threshold = chromosome wide LOD threshold for type I error rates of 20% and 5%; %var = proportion of the total phenotypic variance explained by the QTL; KW = Kruskal-Wallis significance level, given by the P value (1 = 0.1, 2 = 0.05, 3 = 0.01; 4 = 0.005; 5 = 0.001; 6 = 0.0005; 7 = 0.0001). Complete data are referred to 2003 (2002 in case of QTL lack in 2003, as indicated by an asterisk), yearly details (year 2002, 2003 and 2004 are respectively in first, second and third position) are given for LOD scores, percentage of explained variance and Kruskal-Wallis significance, which represent the most variable data

#### Phenology

Ripening-related QTLs were previously reported by [44] on LGs 7, 17 and 18 and by [53] on LGs 7 and 8. In our experiment the phenology sub-traits resulted under the control of three main regions, which are localized on LGs 2, 6 and 16.

On LG2 we identified, reproducibly in the three maps and years, QTLs for flowering time (explaining 7.3–16.4% of total variance), veraison time (explaining 5.8–12.6% of total variance), veraison period (explaining 15.8–44.2% of total variance), flowering-veraison interval (explaining 12.6–21.4% of total variance) and veraison-ripening interval (explaining 14.6–21.7% of total variance). The 1-LOD confidence interval of the QTL for flowering-veraison interval partially overlapped to the confidence interval of the QTL for veraison time, while the 1-LOD confidence interval of the QTL for veraison-ripening interval partially overlapped to the confidence intervals of the QTLs for flowering time (in 2003 and 2004) and veraison time (in 2002). These results reflect the positive correlation observed between flowering-veraison interval and veraison time and the less clear relationship between veraison-ripening interval and flowering/veraison time (Table 4). On the contrary, the 1-LOD confidence intervals of the QTLs for flowering time, veraison time and veraison period were strictly contiguous but not overlapping, thus suggesting the existence of distinct QTLs.

On LG6 of the three maps we detected QTLs for flowering time (13.4–20.8% of total variance, 3 years), veraison time (9.0–9.9% of total variance, 2 years), ripening date (10.2–17.2% of total variance, 2 years), flowering-veraison interval (8.2–8.5% of total variance, 2 years) and flowering-ripening interval (9.1–15.3% of total variance, 2 years). Again, the contiguous but non-overlapping confidence intervals of the QTLs for flowering time, veraison time and ripening date seem to suggest the existence of distinct QTLs, while – not surprisingly based on the correlation observed between these traits – the QTL for flowering-veraison interval coincided with that for veraison time and the QTL for flowering-ripening interval co-localized with that for ripening date.

LG16 turned out to be involved only in the control of veraison, as revealed by the existence in the three maps and years of two coincident QTLs for veraison time (21.1–45.4% of total variance) and flowering-veraison interval (15.4–37.2% of total variance).

Finally, two additional QTLs for flowering time, respectively explaining 6.2–13.9% and 6.3–11.7% of the total phenotypic variance, were found on LG1 in the three maps and years and one additional QTL for veraison-ripening interval, explaining 9.0–16.8% of the total phenotypic variance, was detected on LG12 in two years in the three maps.

No QTL could be identified for flowering period.

#### Berry size and seed content

QTL detection for berry size and seed content was previously reported by [42-44] and [53]. Our results confirm the existence of a major effect QTL on LG18, which was already found by [42] (for berry weight-BW, seed number-SN, seed total fresh weight-STFW, seed total dry weight-STDW, seed mean fresh weight-SMFW, seed mean dry weight-SMDW and seed dry matter-SDM), [43] (for berry weight-BW18a, seed fresh weight-SFW18a and seed number-SN18) and [44] (for berry weight-W25, mean berry size-MBS, number of seeds and seed traces-S&R, number of fully developed seeds-SED and total fresh weight of seeds or seed traces-TFW). The same region was identified in our paternal and consensus maps for three years and explained a great proportion of the phenotypic variance for mean berry weight (27.2–43.1%), percentage of seed dry matter (86.5–91.4%, only in Big Perlon), mean seed fresh weight (13.8–27.5%) and mean seed dry weight (49.3–75.0%). As expected, it coincides with the seedlessness gene *SdI*. The QTLs for berry size and seed content co-positioned on LG18, as already observed by [42,43] and [44]. Unlike [42] and [43], we did not find any evidence for the presence of two distinct QTLs on LG18. Besides this QTL, we detected in three years two significant regions for mean berry weight on LGs 1 (4.6–17.5% of total variance) and 12 (5.1–11.8% of total variance) in the paternal and consensus maps, while other authors identified – in most cases in one or two years – additional QTLs on LGs 1 [44], 5 [53], 11 [42], 13 [53], 14 [44], 15 [43,44]. Our QTL on LG1 does not coincide with that reported by [44] on the same LG.

For seed number we found one QTL on LG2 of the three maps in two years, which explained 19.6–22.9% of the total phenotypic variance. Previous works reported, in addition to the major QTL on LG18, QTLs for this trait on LGs 4 [43], 8 [42], 14 [43,44], 15 and 16 [44], which could be detected in no more than two seasons.

For mean seed fresh weight, in addition to the major QTL on LG18, we identified QTLs on LG 6 (3.5–21.4% of total variance), LG10 (7.7–36.3% of total variance), LG13 (7.2–15.7% of total variance) and finally LG15 (6.7–13.0% of total variance). Other authors found QTLs for this trait on LGs 1, 3, 10, 14 [43], 15 and 16 [44]. Interestingly, our QTL on LG10 for mean seed fresh weight co-localizes with the QTL for the same trait which was detected on LG10 of Dominga × Autumn seedless map [43] and our QTL for mean seed fresh and dry weight on LG15 likely coincides with the QTL identified on the same LG by [44] for number of fully developed seeds and total fresh weight of seeds or seed traces.

For mean seed dry weight, besides the major QTL on LG18, we found two additional QTLs: the first on LG2 co-localizing with the QTLs for flowering time/veraison-ripening interval/mean seed number and explaining 3.7–10.8% of the total phenotypic variance, the second on LG15 co-localizing with the QTL for mean seed fresh weight and explaining 3.1–10.8% of the total phenotypic variance.

## Discussion

In this work we developed genetic maps covering most of the genome for a *Vitis vinifera *cross between two table grape varieties. These maps were used to carry out QTL detection for ripening time, berry size and seed content.

### QTL analysis reliability

When performing interval mapping we verified that QTLs had LOD values higher than linkage group thresholds in more than one growing season. The use of cofactors in multiple interval mapping enabled additional QTLs to be found with respect to simple interval mapping. It was particularly evident in the case of seedlessness-related traits, for which a large part of the total phenotypic variation was explained by the main QTL on LG18. Although MQM is expected to be more powerful, we also used the non-parametric Kruskal-Wallis method in order to confirm that QTLs detected with interval mapping were not artefacts due to large gaps, segregation distortion or non-normal distribution of traits.

As already reported by [42,43] and [44], the QTLs for berry size and seed content co-positioned on LG18. Co-localization of QTLs for other traits was found as well. In most cases it reflected the observed correlation between subcomponents of the same character (i. e. FT and V-R, VT and F-V on LG2; VT and F-V, R and F-R on LG6; MSFW and MSDW on LG15; VT and F-V on LG16; % SDM, MSFW and MSDW on LG18). Nevertheless, we also noticed co-positioning of QTLs for different traits, i. e. on LG2 for flowering time, mean seed number and mean seed dry weight and on LG18 for mean berry weight and seed weight. Based on the known relationship between the gibberellins produced by seeds and berry growth, it has already been suggested that the correlation between berry weight and seedlessness subtraits observed at both phenotypic and genetic level might be due to pleiotropy rather than to tight linkage. Interestingly, two QTLs (on LG1 and 12 in our progeny) have been shown to regulate berry weight without affecting seedlessness, as already reported by other authors on LG1 [44], LG11 [42] and LG15 [43]. These QTLs, along with those specific for seed content identified on LGs 2, 6, 10, 13 and 15, might allow to dissociate the unfavourable correlation between berry size and seedlessness in breeding programs. Similarly, the correlation between flowering time and seedlessness traits that we observed at the genetic level on LG2 could be due to the known effect of gibberellins on flowering. On the contrary, the observed phenotypic correlation between veraison time and seedlessness traits was not supported at the molecular level by coincident QTLs on LG18 as reported by [44]. This might indicate that the genes controlling the two traits function independently of each other, but further confirmation is needed.

General reliability of our results was supported by 1) similar findings in other segregating populations (i. e. the QTL for berry weight and seedlessness subtraits on LG18 [42-44] and the QTLs for mean seed fresh weight on LG 10 [43] and LG15 [44]), 2) Kruskal-Wallis analysis, which revealed significant associations between single marker genotypes and raw phenotypic data, 3) QTL stability over 3 years despite a large year effect. In some cases minor QTLs were detected only in a single year. This might be due to year effects and/or to genotype × year interactions or alternatively to a limited detection power because of the combination of a moderate population size with at least one major QTL responsible for most of the phenotypic variance.

### Marker assisted selection

Some SSR markers co-localized with QTLs and were significantly associated with the corresponding traits in Kruskal-Wallis analysis (Table 5): VMC1E11 (veraison time, flowering-veraison interval), VMC7F2 (mean berry and seed weight), VMC7G3 (veraison period) and VVIB23 (flowering time, veraison-ripening interval and mean seed number). Their usefulness in marker-assisted selection is worth to be tested, as already suggested by [43] and [44] for the markers VMC7F2 and VMC7G3.

### Candidate gene approach

QTL analysis indicates regions of a genome, which contribute to trait variation. The following step is to narrow down these regions to the point where the effects can be ascribed to specific genes. To this purpose we adopted the candidate gene approach [56] at two levels.

First, some "functional candidate genes" selected according to their hypothetical biological function were mapped [see [39] for mapping details]. They encode transcriptional factors influencing flowering time and seed development (EMF, FIE, FIS, GAI) or enzymes involved in the biosynthesis of gibberellins [57], which are known to inhibit floral meristem production, promote seedlessness and increase berry size in grapevine. The EMF (EMBRYONIC FLOWERING) protein has the role to prevent plants from immediately flowering after germination [58]. The FIE (FERTILIZATION-INDEPENDENT ENDOSPERM) protein functions to suppress endosperm development until fertilization occurs [59]. The products of the FIS (FERTILIZATION-INDEPENDENT SEED) genes are likely to play important regulatory roles in seed development after fertilization [60]. Finally, the GAI (GA insensitive) protein negatively regulates GA response [61]. Association analysis revealed a relationship between maize *GAI *homologue (*Dwarf8*) polymorphisms and flowering time [62].

The grapevine homologue (*VvGAI1*) was found to have an effect on flower development as well [24]. We were able to localize onto our maps markers corresponding to *FIE*, *GAI*, *gibberellin 20-oxidase *and *gibberellin 2-oxidase*. *EMF*, *FIS *and the remaining genes involved in the biosynthesis of gibberellins could not be mapped because of lack of homologous grapevine sequences in public databases or amplification failure. The markers corresponding to *FIE *and *gibberellin 2-oxidase *did not co-localize with any QTL, while the position of *GAI*, which was mapped by synteny from the Moscato bianco × *Vitis riparia *progeny, needs to be defined more precisely in order to establish its relationship with the QTLs for flowering time on LG1. Finally, the marker corresponding to *gibberellin 20-oxidase *co-localized with the QTLs for veraison time and flowering-veraison interval detected on LG16 in the three years, but it was not significantly associated with these traits in Kruskal-Wallis analysis.

Second, we used the publicly available genomic sequence of Pinot noir [38] to identify "positional candidate genes" in the proximity of the SSR markers underlying QTLs (Additional file 1). This approach could be applied to all the selected microsatellites except VVIN31 (associated with flowering time) because of contig assembling inconsistencies. Gene prediction was based both on *Vitis vinifera *(as reported in the table) and *Arabidopsis *known splicing sites. A general tendency towards a greater number of smaller genes was observed when referring to *Arabidopsis*, but in most cases results were consistent. Hereafter we discuss the most interesting findings.

QTL analysis suggested an association between the microsatellite VVIB23 and flowering time, flowering-veraison interval, mean seed number and mean seed dry weight. This marker was located in contig AM440415.1 within a predicted gene for a YABBY-like transcription factor. The primary function of *YABBY *gene family members is to specify abaxial cell fate in lateral organs produced by apical and flower meristems [63]. In addition they have been shown to have a role in growth by promoting cell division [64] and in flower formation and development by controlling floral meristem and organ identity [65-67]. Finally, based on their transcriptomic analysis in the *fleshless berry *(*flb*) mutant, [68] attributed to *VvYAB2 *an involvement in early morphogenesis of grapevine berry. They observed for this gene a low and non-differential expression before anthesis, a strong increase after anthesis, which reached the maximum value in the fruit.

The microsatellite VMC2H4 underlying the QTL for veraison-ripening interval was positioned on contig AM486664.1 within a gene for a conserved hypothetical protein and, more interestingly, in the proximity of a gene (*grip31*) encoding a putative ripening-related *Vitis vinifera *protein [Davies and Robinson, unpublished]. The non-coincident position of VMC2H4 with this gene could explain its moderate significance in Kruskal-Wallis analysis.

Finally, the microsatellite VMC7F2 was mapped 0.8 cM far from the seedlessness gene *SdI*. It turned out to be associated with mean berry weight, percentage of seed dry matter, mean seed fresh weight and mean seed dry weight and was located on contig AM464881.2, very close to the predicted gene for *Vitis vinifera *MADS-box protein 5. It is well known that the MADS-box family members have a key role in flower and fruit development. Boss et al. [7] analyzed the expression pattern of this and three other MADS-box genes during grapevine inflorescence and berry development. Based on its female flower carpel-specific expression and its homology with genes of known function, they suggested for *VvMADS5 *a role in ovule and seed development.

As regards the remaining microsatellites reported in Additional file 1, VMC1E11 (underlying the QTLs for veraison time and flowering-veraison interval) was located within a gene encoding a putative protein kinase, VMC5G7 (associated with flowering-veraison interval) within a predicted gene for a heat shock factor, whereas VMC2C10.1, VMC4G6 and VMC4H5 could not be associated to any protein of known function. We expect that the upcoming annotation of grapevine genome will contribute to fill this lacking information.

## Conclusion

In this work we identified the genetic determinants of berry and phenology-related traits in a table grape cross. Three main QTLs on LGs 2, 6, 16 were found to control several subtraits of ripening time, while two additional regions on LGs 1 and 12 turned out to affect only specific phenological characters. A major QTL was detected on LG18 for berry size and seed content, as well as minor QTLs on LG 1, 12 for berry weight and 2, 6, 10, 13, 15 for seed number and weight. The identification of molecular markers closely associated to the main observed QTLs represents a first step towards the design of a marker-assisted program for table grape improvement and encourages to test the role of some positional candidate genes in trait variation.

## Methods

### Plant material

The mapping population utilized in this study (163 individuals) is a random subset of a F_1 _progeny obtained in 1995 from the cross between the table grape cultivars Italia (Bicane × Muscat of Hamburg) and Big Perlon ((Almeria × Cardinal) × Perlon). They have been grown in the field since 1999 at the Experimental Station of the University of Bari (Italy). This population segregates for several agriculturally important traits (phenology, yield, berry size, seed content and Muscat aroma).

### DNA extraction

Genomic DNA was extracted from young leaves and shoot tips after the CTAB method described in [46].

### Molecular marker development and analysis

The progeny was genotyped for 112 SSRs, only partly published [69-78]. Many of them were developed within the Vitis Microsatellite Consortium (VMC) coordinated by AgroGene S. A. (Moissy Cramayel, France). Seventy-two out of the analyzed loci belong to a common set of 86 highly polymorphic and well-distributed SSRs matching the homologous linkage groups of 13 table grape varieties [79]. Additional microsatellite markers were selected based on the available polymorphism and map position information in order to fill gaps and join linkage groups. PCR amplifications were performed in 12.5-μl reactions consisting of 20 ng template DNA, 0.5 μM of each primer, 25 μM of each dNTP, 1.25 μl 10× PCR buffer, 0.5 unit AmpliTaq Gold DNA polymerase (Applied Biosystems, Foster City, CA, USA) and 1.5 or 2 mM MgCl_2 _solution. Amplification protocol was the following: 7 min at 94°C; 35 cycles of 45 sec at 94°C, 45 sec at 56°C and 1 min and 30 sec at 72°C; 7 min at 72°C. Primers failing to amplify at 56°C were further tested at different annealing temperatures. Amplification products were separated either on denaturing 7.5% polyacrylamide sequencing gels (7.5 M urea, 0.5× TBE buffer) with a 2–3 h run at 60 W and visualization by silver staining with a commercial kit (Promega, Madison, Wis., USA) or by capillary electrophoresis in an ABI PRISM 3100 Genetic Analyzer (Applied Biosystems).

AFLP markers were generated after [80]. Primer labelling was performed with [γ-^33^] ATP. Selective amplification assays were carried out with 20 primer combinations over the mapping population. PCR products were separated on 6% denaturing polyacrylamide gels (7.5 M urea, 0.5× TBE buffer) run at 80 W constant power for 2 h 40'.

EST-derived markers were developed after selecting a number of genes based on predicted functions and gene ontologies and revealing molecular polymorphisms through SSCP analysis or minisequencing, as described in [39].

The progeny was also genotyped for the SCAR marker SCC8, proposed by [18] to assist the selection of seedless cultivars.

Berry colour and seedlessness (*SdI*) were scored and mapped as qualitative characters. Black, blue, purple or red were registered as the presence of coloration, yellow or green as absence, as reported in [42]. Pink berries were not present. Seeds and seed traces were classified according to [17]; class 4 (normally developed seeds with totally sclerified integuments) corresponded to presence of seeds, classes 1–3 (only seed traces with unsclerified or partially sclerified integuments) to absence. Completely seedless individuals were not present.

### Map construction

Genotypes with more than 10% missing data were not considered for linkage analysis. Linkage analysis was carried out with JoinMap 3.0 [81]. The only segregations that could not be handled directly by JoinMap (abxa0 and a0xab, where 0 represents a null allele) were included in a duplicated form, as described in [42]. They were treated as two separate loci, one segregating only in the one-banded parent and the other one segregating only in the two-banded parent. The segregation of each marker was tested for goodness-of-fit to the expected segregation using a χ^2 ^test. We decided to keep the distorted markers unless they were of low quality or they significantly affected the order of their neighbours. Linkage groups were determined using threshold values of 5.0 for LOD and 0.45 for recombination rate; the Kosambi mapping function [82] was used for the estimation of map distances. When three rounds of mapping were performed the second-round map was chosen, except in a few cases where the order of markers in the third-round map was confirmed by other mapping experiments reported in literature. Codominant markers and doubly heterozygous dominant markers were used to integrate the homologous pairs of the parental maps into a consensus map. Female, male and consensus maps were aligned using the software MapChart [83].

### Comparison of male and female recombination rates

To compare recombination rates between Italia and Big Perlon, new parental maps were constructed based on 58 common markers. For these markers two data sets were prepared: one in which the maternal parent was coded as homozygous and the paternal parent was coded as heterozygous and a second data set in which the coding was reversed, as described in [48] and [51]. Marker order was fixed according to the original parental maps. A total number of 71 pairs of linked markers were considered. Two point estimates of recombination and LOD scores were supplied by JoinMap for each marker pair in both parents. Mean recombination frequencies with their error values were calculated for each parent in Excel. A genome-wide test for differences in mean maternal and paternal recombination rates was performed using a Z test for comparisons between two populations means. The "Heterogeneity test" function in JoinMap was used to identify, according to a χ^2 ^test, pairs of common markers showing significant differences in recombination frequencies between the two parents.

### Genome length and map coverage

The estimation of genome length was carried out using the method of moment estimator, G_e _= N(N-1)X/K [84], where N is the number of markers, X is the maximum observed map distance between marker pairs above a threshold LOD Z, 5 in this study [85], and K is the number of locus pairs having LOD values at or above Z. The confidence interval was computed according to [86] from the equation I_α_(G_e_) = G_e_(1 ± n_α_K^-1/2^)^-1^, where n_α _= 1.96 for an α of 5%. Two estimates of genome map coverage (C_e_) were calculated for each parent: by the equation C_e _= 1-P_1, N _and P_1, N _= 2R/(N+1) [(1-X/2G)^N+1^-(1-X/G)^N+1^]+ [(1-RX/G)(1-X/G)^N ^[54], where R is the haploid number of chromosomes, N is the number of markers and X is the maximum centiMorgan distance when Z = 5, and by the equation C_e _= 1-e^-XN/1.25Ge ^[55]. Finally the observed genome map coverage was the ratio between observed and estimated genome length. In all cases Kosambi map distances were used. The above calculations were first performed using all mapped loci and then excluding all AFLPs.

### Phenotypic evaluation of ripening time, berry weight and seed content

Segregating traits were evaluated in three growing seasons.

Ripening time was analyzed by scoring the following component traits: flowering (FB, FE) and veraison (VB, VE) beginning and end dates and ripening (R) date. Veraison was established according to berry colour and consistency change, while ripening was reached when sugar content of must was approximately 16°Brix. In order to minimize the great variability among the different berries of the same cluster as well as among the berries of different clusters, sugar content values from 3 randomly taken berries per cluster and 2–3 representative clusters per genotype were averaged. From these measures flowering time (FT = date corresponding to 50% opened flowers), flowering period (FP = time between the opening of the first flowers and that of all the flowers), veraison time (VT = date corresponding to veraison of 50% of the berries), veraison period (VP = time between the veraison of the first berries and that of all the berries), flowering-veraison (F-V), flowering-ripening (F-R) and veraison-ripening (V-R) intervals were finally calculated.

For each genotype, 100 berries were randomly taken from a mixture of 2–3 representative clusters and weighted (berry weight, BW); mean berry weight (MBW) was then calculated. All the seeds and seed traces from 25 berries of the mixture were extracted, counted (seed number, SN), weighted (total seed fresh weight, TSFW), dried at 80°C for 48 hours and weighted again (total seed dry weight, TSDW). From these measures mean seed number per berry (MSN), percentage of seed dry matter (% SDM = TSDW/TSFW*100), mean seed fresh weight (MSFW = TSFW/SN) and mean seed dry weight (MSDW = TSDW/SN) were computed.

The normality of each trait distribution was evaluated by the Kolmogorov-Smirnov test. Year effect was tested with analysis of variance and Kruskal-Wallis test. Phenotypic correlations between traits within years and between years within traits were determined using the non-parametric Spearman correlation coefficient. These statistical analyses were performed with SPSS 11.0.

### QTL analysis

QTL detection was carried out on each parental map using the software MapQTL 4.0 [87] and the data from 3 separate years. It was based on two different methods: the non-parametric Kruskal-Wallis (KW) rank-sum test and interval mapping [88]. LOD thresholds at 0.95 and 0.80 significance were established for each linkage group through 1000 permutations [89]. Simple interval mapping (SIM) analysis was initially performed to find regions with potential QTL effects and then scored markers in those regions were used as cofactors in multiple QTL models (MQM analysis). When a new QTL was found this way, markers linked to this QTL were added as cofactors and the search was reiterated until no new QTL could be detected. QTL position was estimated from the location of the maximum LOD value and a 1-LOD support interval.

The complete sequence of the SSR markers underlying the main QTLs was used to identify by alignment (BLASTN) the surrounding genomic sequence of Pinot noir clone ENTAV115 [38]. In each case contigs were selected based on the following criteria: e-value < e^-20^, aligned sequence length > 100 nucleotides, identity > 90%. SSR and contig nucleotidic sequences were aligned through MEGA3 software [90]. Putative genes in the genomic DNA were predicted by means of the software FGENESH [91]. Protein homologies of the coding regions were searched against NCBI NonRedundant Protein database [92] with BLASTP. Protein subcellular localization was predicted by using the softwares Predotar 1.03 [93] and SignalP 3.0 [94].

## List of abbreviations

% SDM: percentage of seed dry matter; AFLP: Amplified Fragment Length Polymorphism; cM: centiMorgan; ER: endoplasmic reticulum; EST: Expressed Sequence Tag; F-R: flowering-ripening interval; FT: flowering time; F-V: flowering-veraison interval; GA: gibberellic acid; LG: linkage group; LOD: logarithm of odds; MBW: mean berry weight; MQM: multiple QTL mapping; MSDW: mean seed dry weight; MSFW: mean seed fresh weight; MSN: mean seed number; nt: nucleotide; QTL: Quantitative Trait Locus; R: ripening date; SCAR: Sequence Characterized Amplified Region; SSCP: Single Strand Conformation Polymorphism; SSR: Simple Sequence Repeat; VP: veraison period; V-R: veraison-ripening interval; VT: veraison time.

## Authors' contributions

LC carried out the mapping of microsatellites, the statistical and bioinformatic analyses, participated in the phenotypic evaluation and drafted the manuscript. JB developed candidate gene markers and participated in the phenotypic evaluation. FL carried out AFLP analysis and participated in the phenotypic evaluation. GF produced the cross and coordinated the field studies. MSG conceived and coordinated the study, and revised the manuscript. All authors read and approved the final manuscript.

## Supplementary Material

Additional file 1 Genomic sequence underlying QTLs. Mean features of the Pinot noir genomic contigs that align with SSR markers underlying QTLs: number, length, predicted genes and proteins. Click here for file
