# Supplementary material for: Berry and phenology-related traits in grapevine (Vitis vinifera L.): From Quantitative Trait Loci to underlying genes
Source: BMC Plant Biol. 2008 Apr 17;8:38. doi: 10.1186/1471-2229-8-38 (PMC2395262; doi:10.1186/1471-2229-8-38)
Supplement: Additional file 1 — Genomic sequence underlying QTLs. Mean features of the Pinot noir genomic contigs that align with SSR markers underlying QTLs: number, length, predicted genes and proteins. [file 1471-2229-8-38-S1.pdf]

Additional files

Additional file 1 - Mean features of the Pinot noir genomic contigs [38] that align with SSR markers underlying QTLs: number, length, predicted genes and proteins

SSR	LG	Trait	Contig number	Contig length (nt)	SSR position within contig	Predicted gene position	Chain	Predicted protein length (aa)	Protein similarity	Subcellular localization prediction
**VMC5G7**	2	F-V	AM426260.2 (CAAP02000071.1)	26662	14770-15156	**<u>2488-15183</u>**	**-**	**590**	**Heat shock factor protein hsf8-related [*Glycine max*, AAS15800]**	**None**
						17802-25590	+	1576	Gag-pol polyprotein-like [*Solanum tuberosum*, AAU89779]	None/Plastid
**VMC2C10.1**	2	VT, V-R	AM437882.2 (CAAP02000034.1)	42409	19777-20190	<u>25-12480</u>	-	616	COG6 domain (Golgi complex)	None/Possibly mitochondrial
						**<u>17532-24691</u>**	**+**	**220**	**Unknown protein**	**None**
						25695-26921	-	117	Unknown protein	None/Possibly plastid
**VVIB23**	2	FT, V-R, MSN, MSDW	AM440415.1 (CAAP02004239.1)	64774	38192-38617	3495-6457	+	393	Trehalose-phosphate phosphatase [*Nicotiana tabacum*, AAU03490]	None
						7743-17562	+	384	Transaldolase ToTAL2 [*Lycopersicon esculentum*, AAG34725]	Signal peptide/Plastid

						18898-23013	+	661	Vacuolar invertase 2, GIN2 [*Vitis vinifera*, AAB47172]	Signal anchor/None
						26576-31917	+	560	Copper ion binding [*Arabidopsis thaliana*, NP_192979]	Signal peptide/ER
						**37627-43729**	**-**	**272**	**YABBY-like transcription factor GRAMINIFOLIA [*Antirrhinum majus*, AAS10177]**	**Signal anchor/ER**
						47472-51589	+	307	Pentapeptide domain Ricin-type beta-trefoil domain	Signal anchor/Plastid
						51769-53143	+	160	Pentapeptide domain	None/Plastid
						53159-55272	-	350	No similarity	None
						56263-58465	-	614	Gag-pol polyprotein [*Vitis vinifera*, AAF20282]	None
						62047-64593*	-	574	Subtilisin-like protease [*Glycine max*, AAQ23176]	Possibly mitochondrial
**VMC4G6**	6	VT, F-V	AM454821.2 (CAAP02000198.1)	41067	28363-28653	*313-6492	+	688	Unknown protein	None
						6612-8295	-	246	Integrase, catalytic region [*Medicago truncatula*, ABE91551]	None
						8379-16891	-	1242	Reverse transcriptase domain Integrase core domain	None
						17009-19101	+	129	Unknown protein	None

						**19461-28624**	**+**	**271**	**Unknown protein**	**None**
						30029-35739	+	280	Unknown protein	None
						35935-40355*	-	1144	Integrase core domain containing protein [*Solanum demissum*, ABI34329]	None
**VMC4H5**	6	R, F-R	AM438219.1 (CAAP02000936.1)	20473	17012-17476	6882-9822	+	574	Trehalose-6-phosphate synthase [*Ginkgo biloba*, AAX16014]	None
						12371-16815	+	238	Unknown protein	None
**VMC2H4**	12	V-R	AM486664.1 (CAAP02001022.1)	19711	7984-8412	1881-3089	+	173	Putative ripening-related protein [*Vitis vinifera*, CAB85626]	None
						**7347-9086**	**+**	**164**	**Conserved hypothetical protein [*Medicago truncatula*, ABE79920]**	**None**
						10845-17166	-	1449	Retrotransposon gag protein domain Reverse transcriptase domain Integrase core domain	None
**VMC1E11**	16	VT, F-V	AM445210.1 (CAAP02001047.1)	32114	26432-26825	817-3807	-	530	Serine/Threonine protein kinase catalytic domain	Signal peptide/ER
						5671-8861	+	547	Tyrosine kinase catalytic domain	Signal peptide/ER
						18105-19513	+	219	DUF1442 domain (unknown function)	Plastid

						**22983-28610**	**-**	**798**	**ATP binding / protein kinase / protein serine/threonine kinase / protein-tyrosine kinase [*Arabidopsis thaliana*, NP_199969]**	**None**
**VMC7F2**	18	MBW, % SDM, MSFW, MSDW	AM464881.2 (CAAP02001498.1)	28046	24475-24950	*1555-9683	+	538	Elongation factor 1-gamma [*Prunus avium*, Q9FUM1]	None/Possibly plastid
						25131-27824	+	85	MADS-box protein 5 [*Vitis vinifera*, AAM21345]	None

The predicted genes specifically containing the SSR sequences are in boldface, those with suspect extremely long introns (more than 3000 nt) are underlined, those lacking the transcription start site or the polyA sequence are marked with an asterisk. When two subcellular localization predictions were reported, the first one was obtained with SignalP 3.0 [94], the second one with Predotar 1.03 [93].
In brackets are reported contigs identified after BLASTN alignment against the Whole-Genome Shotgun reads (WGS) database [37]
